# Autoimmune Myopathies: Where Do We Stand?

**DOI:** 10.3389/fimmu.2016.00234

**Published:** 2016-06-14

**Authors:** Jean-Philippe Simon, Isabelle Marie, Fabienne Jouen, Olivier Boyer, Jérémie Martinet

**Affiliations:** ^1^Laboratory of Neuropathology, CHU Caen, Normandie University, UNICAEN, Caen, France; ^2^Normandie University, UNIROUEN, Pathophysiology and Biotherapy of Inflammatory and Autoimmune Diseases, INSERM, CHU Rouen, Rouen, France

**Keywords:** myositis, necrotizing myopathy, autoantibodies, inclusion-body myositis, dermatomyositis

## Abstract

Autoimmune diseases (AIDs) as a whole represent a major health concern and remain a medical and scientific challenge. Some of them, such as multiple sclerosis or type 1 diabetes, have been actively investigated for many decades. Autoimmune myopathies (AIMs), also referred to as idiopathic inflammatory myopathies or myositis, represent a group of very severe AID for which we have a more limited pathophysiological knowledge. AIM encompass a group of, individually rare but collectively not so uncommon, diseases characterized by symmetrical proximal muscle weakness, increased serum muscle enzymes such as creatine kinase, myopathic changes on electromyography, and several typical histological patterns on muscle biopsy, including the presence of inflammatory cell infiltrates in muscle tissue. Importantly, some AIMs are strongly related to cancer. Here, we review the current knowledge on the most prevalent forms of AIM and, notably, the diagnostic contribution of autoantibodies.

Bohan and Peter originally classified myositis into two major groups based on clinical, electromyographical, and immunohistological features: polymyositis (PM) and dermatomyositis (DM), the latter associating skin manifestations to muscle weakness (1). Indeed, the presence of a muscle inflammatory infiltrate is a hallmark in autoimmune myopathies (AIM), but it is not discriminating enough since it cannot *per se* identify each AIM subtype and maybe present in some types of muscular dystrophies. Sporadic inclusion-body myositis (sIBM), resistant to steroids and associating both autoimmune and degenerative components, was individualized more recently (2, 3). When defined according to the Bohan and Peter classification, PM was initially considered as the archetype of AIM, although it seems to be an uncommon pathological entity, some experts even arguing that it barely exists (4, 5). Indeed, according to newer, more stringent criteria, most AIM initially diagnosed as PM were reclassified as overlap myositis (OM), a condition with not only musculoskeletal but also extramuscular involvement and/or association to autoantibodies (aAbs) (6). Anti-tRNA synthetase syndrome (ARS syndrome) is the archetype of OM combining myositis to interstitial lung disease, arthritis, Raynaud phenomenon, and mechanic’s hands. Hence, ARS syndrome has been considered by some authors as an atypical clinical form of DM (7). OM and DM are the more frequent AIM (67 and 18%, respectively, according to Troyanov et al.) (6). Around 60% of patients with AIM have myositis-specific aAbs (8), and it is presumable that this frequency will reach higher levels when appropriate diagnostic immunoassays are more widely used and new specificities are discovered (Figure 1). The more recently identified entity is represented by necrotizing autoimmune myopathies (NAM), also sometimes referred to as immune-mediated necrotizing myopathies. This group of severe AIM is characterized by minimal inflammatory infiltrate but predominant necrotic fibers on muscle biopsy (9). The discovery of aAbs directed to components of the signal recognition particle (SRP) (10) or to 3-hydroxy-3-methylglutaryl-coenzyme A reductase (HMGCR) (11, 12) has been instrumental in identifying this entity.

**Figure 1:**
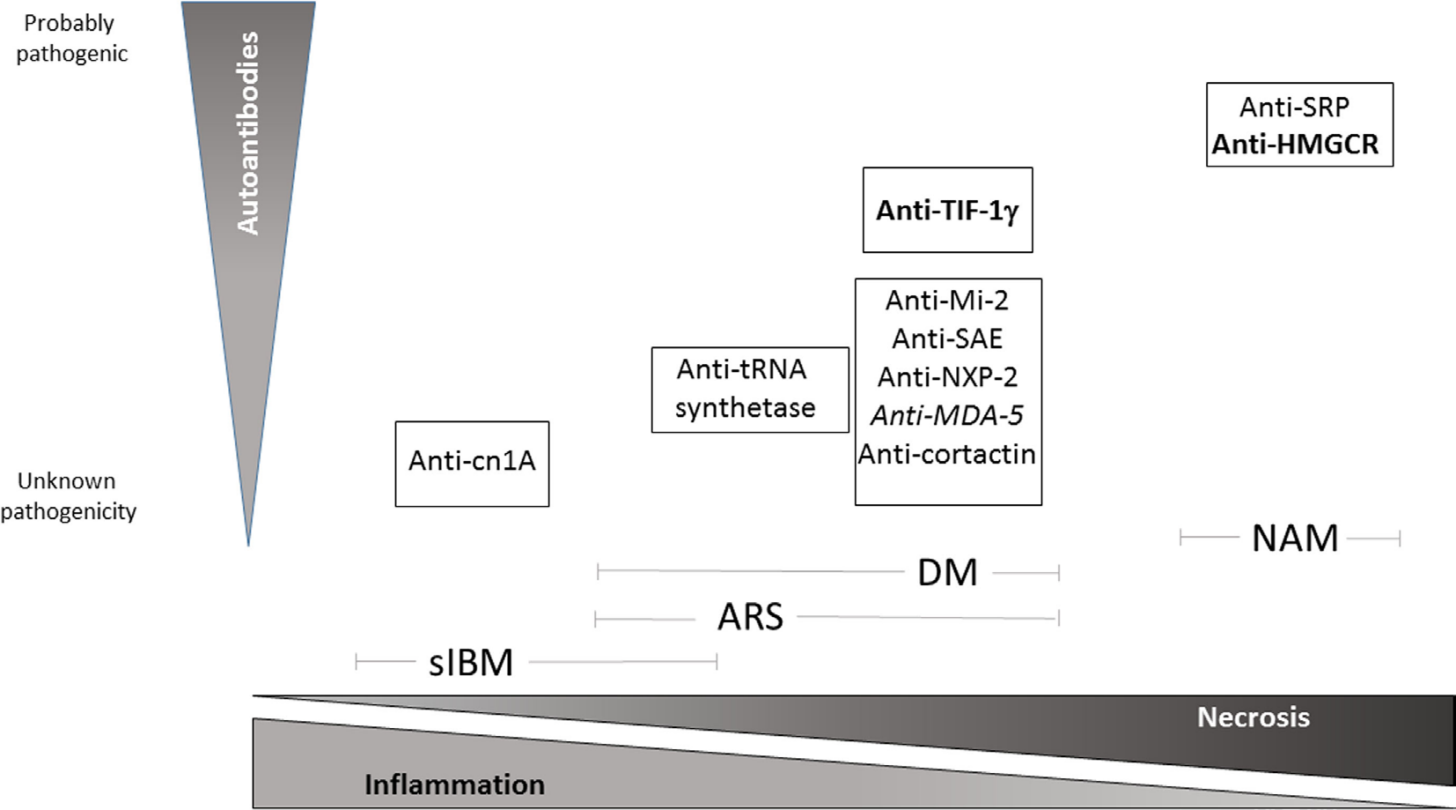
**Schematic view of pathological pattern of inflammatory myopathies and proven degree of pathogenicity of specific autoantibodies relative to each IM subtypes**. Bold: high risk of neoplasia, Italics: amyopathic DM. NAM, necrotizing autoimmune myopathy; DM, dermatomyositis; ARS, anti-t-rna synthetase syndrome; sIBM, sporadic inclusion-body myositis.

## Dermatomyositis and Overlap Myositis

Dermatomyositis affects both children and adults, most frequently women, and is associated with cancer in adults and calcinosis in children. The clinical presentation of DM includes skin manifestations that usually precede muscle weakness (13). Skin features are varied and some are very specific such as a violaceous eruption (Gottron’s rash) on the knuckles which may evolve into a scaling discoloration (Gottron’s papule), purple periorbital heliotrope rash with edema notably on upper eyelids, while others are less specific as erythematous rash on the face, knees, elbows, malleoli, neck, anterior chest (in a V-sign), back and shoulders (in a shawl sign), or periungeal erythema, painful to pressure (manicure sign) (14). Muscle weakness constantly involves limb girdle muscles and less frequently respiratory and pharyngeal muscles (15).

**Figure 2:**
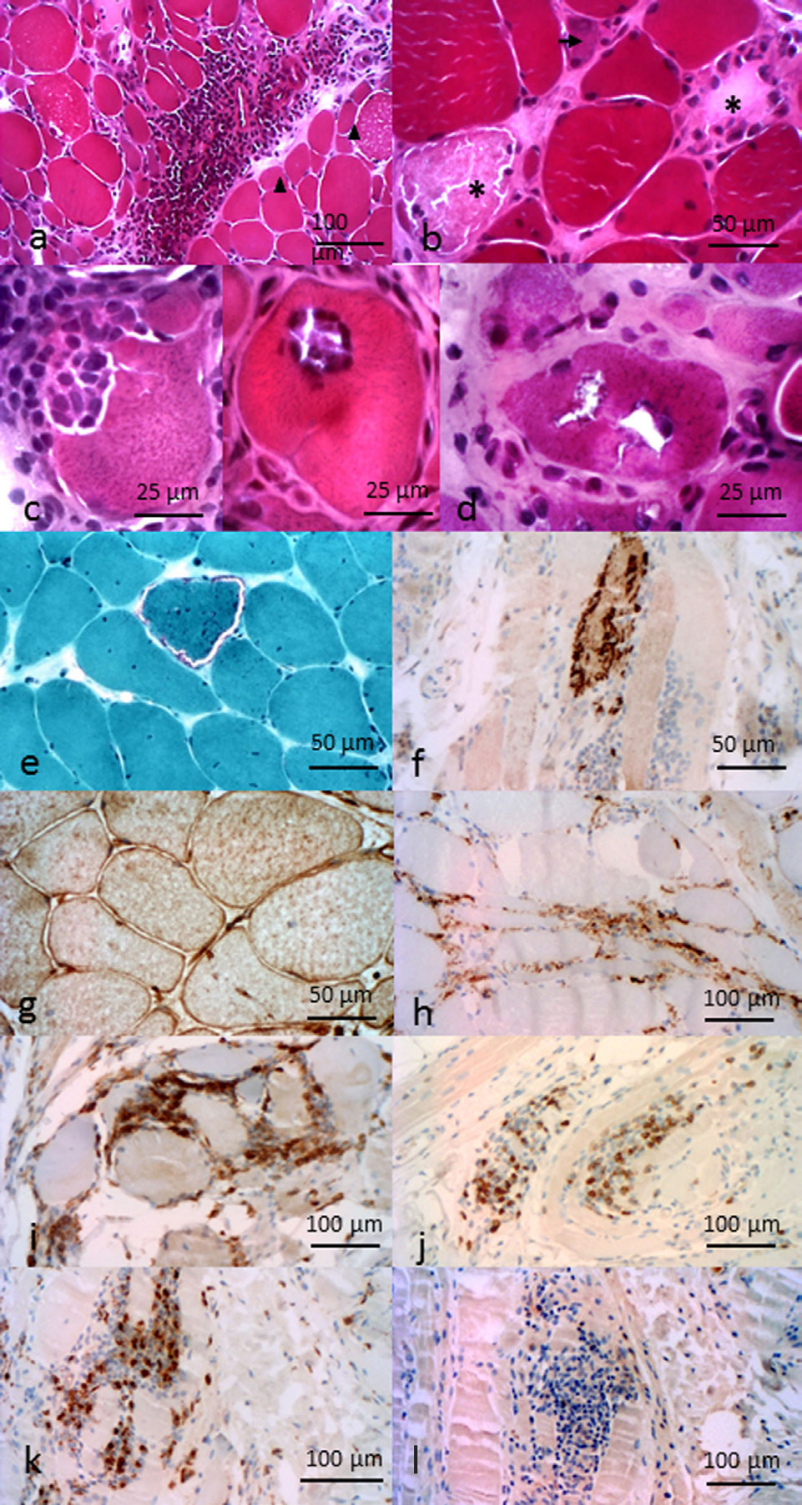
**Pathological features of inflammatory myopathies**. Pathological features of DM. **(A)** Perimysial and perivascular inflammatory cell infiltrate. Perifascicular myofiber atrophy (arrow heads). Pathological features of anti-HMGCR NAM. **(B)** Necrotic (asterisk) and regenerative (arrows) myofibers, sparsely infiltrate pathological features of sIBM. **(C)** Invaded fibers by inflammatory cells. **(D)** Rimmed vacuoles. **(E)** Ragged-red fiber. **(F)** P62 aggregates. **(G)** Immunohistochemichal evidence of MHC-I myofibers expression. **(H)** C5b–9 membrane attack complex in capillaries. Composition of the endomysial inflammatory infiltrate **(I)** CD4^+^ cells, **(J)** CD8^+^ cells, **(K)** CD68^+^ cells, and **(L)** rare CD20^+^ cells. All features provided by Dr. Jean-Philippe Simon.

As a consequence of myofiber injury, creatine kinase is released and found elevated in the serum. Electromyography and MRI may also be useful to evaluate disease topography and guide muscle biopsy. In the presence of typical skin manifestations, muscle biopsy is not always performed. When it is the case, histological analysis establishes the diagnosis by showing features that, in addition to inflammation and myofiber necrosis plus regeneration, distinguish DM from other myositis pattern: endomysial microangiopathy with membrane attack complex (C5b–9) capillaries staining and ischemic fiber lesions. Also, in DM, mononuclear cells composed of macrophages, B lymphocytes, and plasmacytoid dendritic cells (pDC) predominantly infiltrate perivascular and perimysial areas (Figure 2A), whereas normal muscle fibers do not express detectable levels of HLA class I molecules (16, 17), sarcolemmal re-expression of HLA class I is a hallmark of most types of myositis, with a perifascicular enhancement, in the case of DM (15). Recently, a lymphoid follicle variant of juvenile DM has been described and is associated with a severe prognosis (18).

Several aAbs are associated with different clinical forms of DM and have an impact on the prognosis (19). Anti-Mi2 aAbs are positive in 10–20% of DM and associated with a low risk of associated cancer and a better prognosis (20). Anti-Mi2 aAbs are directed against the nucleosome remodeling histone-deacetylase (NURD) nuclear protein complex, implicated in DNA transcription. In contrast, anti-TIF1γ aAbs are positive in more than 10% of DM and the presence of this aAb is highly associated with cancer (21). As in other DM, transcription intermediary factor 1 (TIF1) family proteins, a nuclear transcription factor (22), is implicated in the TGFβ pathway (23). Anti-melanoma differentiation-associated gene 5 (anti-MDA-5) aAbs recognize an innate immune protein implicated in antiviral response and is positive in about 10–20% of DM (24). They are more frequent in amyopathic forms of DM, with severe interstitial lung disease and ulceral lesions (25). Anti-NXP-2 aAbs are more frequent in juvenile DM and are associated with calcinosis. Others, such as anti-small ubiquitin-like modifier activating enzyme (anti-SAE) (26, 27) or anti-cortactin (28), are currently found more rarely and also less routinely sought for.

The physiopathology of DM is not well understood (13, 29). Muscle biopsy shows not only immune cells infiltrates but also C5b–9 deposits. Since DM is strongly associated with the presence of aAbs, it is presumable, yet not clearly demonstrated, that these aAbs may activate complement triggering the release of pro-inflammatory cytokines, upregulation of adhesion molecules on endothelial cells, and migration of B and CD4^+^ T lymphocytes and pDC (30–32). Type 1 interferons produced by pDC, in turn, stimulate the production of pro-inflammatory cytokines and enhance the expression or HLA class I and class II molecules (33).

Dermatomyositis associated with anti-TIF1γ aAbs is strongly related to cancer (21). One (non-demonstrated) hypothesis is that somatic mutations in cancer cells may have occurred in the TIF1γ gene, yielding to the production of an immunogenic non-self neoantigen, which could lead to autoimmunity after epitope spreading of the B cell response, following the example of POLR3 mutations in systemic sclerosis (34). This hypothesis would imply that anti-TIF1γ aAbs and/or T cells are directly pathogenic, which remains to be demonstrated.

High-dose oral corticosteroids (1 mg/kg) represent the first-line treatment in DM (13, 35). Methylprednisolone bolus (500 mg/day–1 g/day during 3 days) can be considered when severe motor deficit or life-threatening extramuscular manifestations are present. Immunosuppressive treatments are used as second-line treatment (methotrexate, azathioprine) if corticosteroid treatment is ineffective or if the patient has developed serious corticosteroid-related side effects. Others treatment can be considered as third-line therapy (36): intravenous immunoglobulins, ciclosporin, cyclophosphamide, or rituximab.

Anti-tRNA synthetase syndromes are defined by aAbs positivity directed against one aminoacyl-tRNA synthetase enzyme. ARS syndrome has been initially described as a specific clinical presentation regrouping myositis, lung interstitial disease, arthritis, mechanic’s hands, and Raynaud phenomenon. There is some clinical variation depending on the aAb. Patients may initially present with an isolated lung involvement. This syndrome is associated with specific aAb directed against one of the aminoacyl-tRNA-synthetase enzymes that enable tRNA to bind to specific amino acids. Each enzyme is specific for an amino acid/tRNA pair (37). The most common aAbs, by far, recognize histidyl-tRNA-synthetase (anti-JO-1), others less frequently alanyl-tRNA-synthetase (anti-PL-12) or threonyl-tRNA-synthetase (anti-PL-7). The pathological pattern is close to that of DM (38). Recent pathological studies argue for some specific features, such as a HLA-DR expression, with a specific perifascicular pattern (39), as the topography of necrotic myofibers (40), ARS syndrome usually respond to corticosteroids and immunosuppressive drugs. Rituximab can be considered in case of severe muscular weakness or intestitial lung disease (41, 42).

## Necrotizing Autoimmune Myopathies

Although their frequency is difficult to determine precisely at this stage, NAM probably account for 20% of AIM. They mainly affect adults, most frequently women. NAM clinical presentation is characterized by symmetrical and proximal muscle weakness leading to muscle atrophy, which can be associated with interstitial lung disease, dysphagia, or dyspnea (43, 44). Some patients present with progressive, corticosteroid resistant myositis, while other forms may masquerade as a muscular dystrophy, yielding to a delay in diagnosis.

Creatine kinase is generally highly elevated (>4,000 U/L) and correlated with disease severity during follow-up (10, 45, 46). Electrocardiogram may be abnormal (13, 47). Muscle MRI can be useful to confirm muscle involvement, evaluate disease topography, and guide the biopsy.

The prominent histological finding in NAM is the presence of necrotic and regenerative myofibers. Atrophic and irregularly shaped myofibers are also present (Figure 2B). When present, inflammatory infiltrates are mostly composed of macrophages (48, 49). Yet, a mild lymphocytic infiltrate can be found in some patients with presence of CD4^+^ and CD8^+^ T cells, B cells, and dendritic cells restricted to the perivascular and endomysial regions. C5b–9 deposits can be found on necrotic myofibers, sarcoplasm of myofibers, and in some capillaries (50). As opposed to other AIM, HLA class I re-expression is mild and scattered, if present (43).

Necrotizing autoimmune myopathies are strongly associated with specific aAbs in two-third of cases: anti-SRP or anti-HMGCR aAbs. Anti-SRP aAbs mainly, but not exclusively, target the 54 kDa subunit of the SRP complex localized in the cytoplasm or at the surface of the endoplasmic reticulum, when bound to the SRP receptor. SRP allows the transfer of nascent proteins to the endoplasmic reticulum during protein synthesis. Anti-SRP aAbs from patients are able to block this mechanism (51, 52). Younger patients with anti-SRP aAbs may develop a form mimicking a muscular dystrophy (53). Anti-HMGCR aAbs target the catalytic domain of hydroxyl-methylglutaryl-coenzyme A reductase. This endoplasmic reticulum membrane enzyme is involved in the biosynthesis of cholesterol and is the pharmacological target of the hypocholesterolemic drugs, statins (11, 54). We developed quantitative immune-assays based on the ALBIA/Luminex technology, which showed that anti-SRP and anti-HMGCR aAbs levels correlate with disease activity, as evaluated by CK levels and muscle strength (10, 45). One-third of NAM patients still do not exhibit known aAbs, but it is anticipated that new specificities will be discovered.

Necrotizing autoimmune myopathies pathophysiology is still poorly understood, notably because of a lack of animal models. An autoimmune mechanism has been early suggested by the association between NAM and the above-mentioned anti-SRP aAbs (50, 55). It should be reminded that both anti-SRP and anti-HMGCR aAbs target intracellular proteins and the question of how they may reach their cognate antigen remains a mystery. However, the paucity of inflammatory infiltrate, the mild re-expression of HLA class I by non-necrotic and non-regenerative myofibers, and the pathogenic implication of antibodies suggested by the correlation between level of aAbs and disease activity strongly argues for a humoral-related disease with aAb-dependent complement-mediated myofiber attack, eventually leading to myofiber necrosis and regeneration from satellite cells (11, 45, 54, 56). The presence of C5b–9 deposits on muscle cells further supports the hypothesis of a direct role of anti-SRP and anti-HMGCR aAbs. Approximately half of anti-HMGCR positive NAM patients have been exposed to statins, molecule that inhibit activity and provoke overexpression of HMGCR (11, 54). It cannot be excluded that the binding of a statin to HMGCR might also change the conformation of the protein, leading to the generation of new epitopes to which the immune system would not be tolerant (44). We have some experimental evidence suggesting that anti-SRP and anti-HMGCR aAbs are directly pathogenic *in vitro* on muscle cells and *in vivo* in mice.

Currently, there is no validated therapeutic strategy for NAM. High-dose oral corticosteroids (1 mg/kg) are the first line of treatment (47). However, they are not sufficient to control disease and need to be combined with immunosuppressive therapy (methotrexate, azathioprine, and cyclosporine) and intravenous immunoglobulins (44, 57, 58). In resistant patients, due to the presumable pathogenic role of aAbs, plasmapheresis can be helpful and lead to clinical improvement. Also, some studies have shown the interest to treat patients with rituximab (50, 59) or cyclophosphamide (60). A recent study has evidenced an association between anti-HMGCR positive and seronegative NAM to neoplasia, whereas anti-SRP positive NAM was not related to cancer (61). This study also showed that the presence of cancer is a main determinant of outcome. In case of exposure, statin treatment must be immediately discontinued. Statin exposed patients seem to have a better response to treatment (45, 62). Finally, it should be stated that statins may be present in the food (some mushrooms or yeast for instance) and patients could have been exposed without being aware of it. Therefore, absence of statin exposure should not discard this diagnosis.

## Sporadic Inclusion-Body Myositis

Sporadic inclusion-body myositis is the most frequently acquired myopathy in patients over 50 years (63). Its natural course is quite slow with a progressive muscular weakness worsening year after year often leading to a loss of ambulation, respiratory muscle weakness, and dysphagia (3). sIBM affect striated muscle with a characteristic pattern affecting quadriceps, fingers deep flexors, and possibly pharyngeal muscles.

Pathological criteria have been of major importance to retain the diagnosis of sIBM (2). The new criteria no longer require bringing together all the pathological hallmarks (64): they include presence of inflammatory infiltrates (Figure 2C) with mononuclear cell invasion of non-necrotic muscle fibers (partial invasion), vacuolated muscle fibers (Figure 2D), and intracellular amyloid protein deposits or 15–18 nm tubulofilaments in the cytoplasm or the nucleus by electron microscopy. Pathological marks have been historically divided between inflammatory and degenerative signs. Degenerative signs are based on the presence of abnormal accumulation of β-amyloïdogenic deposits (65–67). These deposits are located in the muscle fibers of sIBM patients, whereas they used to be observed extracellularly in Alzheimer’s diseases. Others proteins known to be implicated in degenerative processes have been found, such as ubiquitin (68). The two main pathways of protein degradation, 26S-proteasome (69) and autophagy (70), are impaired, the latter generating rimmed vacuoles. Proteins linked to these pathways are accumulated, as demonstrated by immunostaining, such as p62 or TDP43 (Figure 2F); they represent sensitive pathological markers of sIBM (71, 72). On the other hand, inflammatory infiltrates also accompany these degenerative signs. They are predominantly composed of CD8^+^ T cells and macrophages and a minority of CD4^+^ T cells and CD20^+^ B cells (Figures 2I–L). CD8^+^ T cells and macrophages can invade non-necrotic muscle fibers (73). HLA class I molecules have a diffuse and high overexpression at the sarcolemma (16, 74), typically more intense than in other AIMs (Figure 2G). It is presumable that these HLA molecules can present yet unknown muscle auto-antigens to CD8^+^ T cells. Complement C5b9 membrane attack complex is usually pathologically expressed by capillaries (Figure 2H). Moreover, Pestronk recently identified mitochondrial abnormalities (COX negative and/or ragged-red fibers; Figure 2E) as hallmarks of sIBM pathological pattern (38). Yet, despite these signs of probable autoimmunity, sIBM patients respond poorly to immune treatments (3).

The fundamental question remains as to whether sIBM is primarily an inflammatory or degenerative myopathy. We and others have defended elsewhere the view that the starter of sIBM is more likely to be inflammation within muscle (75). Indeed, there are few arguments to consider that degeneration could be the initial event. The strongest would be the lack of efficiency of immunosuppressive drugs (76–79), although two trials assessing massive immunosuppression have reported a strength stabilization (80, 81). Also, the initially selective muscular pattern (quadriceps and fingers deep flexors) is rather a genetic myopathy trait. It should be mentioned here that hereditary IBM (hIBM) may be secondary to mutation in the valosin-containing protein (*VCP*) (82, 83) or UDP-*N*-acetylglucosamine-2-epimerase/*N*-acetylmannosamine kinase (*GNE*) (84) gene. Yet, hIBM pathological patterns only contain degenerative elements and no immune infiltrates, indicating that muscle degeneration *per se* is probably not sufficient to elicit a myositis process. Alternatively, more arguments can be found to support inflammation as an initial trigger. They include association of sIBM to other autoimmune diseases (AIDs) (85), to aAbs directed against cytosolic 5′-nucleotidase1A (86, 87), or to some HLA alleles (88). Also, exposure of human myotubes to IL-1β causes intracellular aggregation of β-amyloid (89).

## Summary

Autoimmune myopathy represents a group of severe inflammatory diseases. The search for aAbs has substantially improved their diagnosis and may also inform on their prognosis, notably when there is an associated risk of cancer. Studying myositis offers exciting pathophysiological and clinical research perspectives in immunology. Important issues relate to the nature of the association between cancer and adult DM, the pathogenicity of aAbs in NAM, the elucidation of the *primum movens* of sIBM, the identification of new aAbs and therapeutic targets, and the validation of therapeutic protocols using immunosuppressants or biodrugs. Clearly, the development of appropriate animal models will be instrumental in this perspective.

## Author Contributions

OB gave an oral communication on this topic at Immunocolombia 2015 and coordinated the redaction of this mini review. JM and JPS wrote the first draft of this article. JPS designed figures. IM and FJ critically reviewed the manuscript for important intellectual content. All authors approved the final version.

## Conflict of Interest Statement

The authors declare that the research was conducted in the absence of any commercial or financial relationships that could be construed as a potential conflict of interest.
